# Development of a mobile application to assess Brazilian schoolchildren's diet: CADE – food consumption at home and at school

**DOI:** 10.1017/jns.2022.25

**Published:** 2022-04-11

**Authors:** Jade Veloso Freitas, Sandra Patricia Crispim, Marina Campos Araujo

**Affiliations:** 1 Sergio Arouca National School of Public Health, Oswaldo Cruz Foundation, Ministry of Health, Rio de Janeiro, RJ, Brazil; 2 Department of Epidemiology, Institute of Social Medicine, State University of Rio de Janeiro; 3 Department of Nutrition, Federal University of Paraná, Curitiba, Brazil

**Keywords:** Food consumption, Mobile application, Schoolchildren, Technology

## Abstract

The development of technologies for children's dietary assessment shows important potential for reducing the occurrence of inherent errors in traditional methods. The present study aimed to describe the development of a mobile app for the dietary assessment of Brazilian schoolchildren. The mobile app assesses schoolchildren's diet with self-report by their parents or guardians in the home environment, through multiple-pass 24-hour recall coupled with a food propensity questionnaire; and by an adult in the school environment, through a food record. The tool presents a database of food items usually consumed by Brazilian schoolchildren, including modes of preparation, probing foods and types of food quantification such as digital photos of household measurements and food portions. The CADE app (food consumption at home and at school) contains 2125 food items, 9 options for preparation methods and 18 options for probing items. There are 75 options for household measurements, also including 26 digital photos of four types of household measurements and 440 photos of portion sizes of 90 foods from the Brazilian Manual of Child Food Portion Quantification. Some innovative features include an interface to take photos of the child's meals and report seconds and leftover food consumption, besides the possibility of receiving notifications on the mobile device to remember to report the diet. The CADE app can assist the standardisation and automation of dietary data collection from schoolchildren, support food and nutrition data in childhood and promote research in nutritional epidemiology while reducing data collection costs.

## Introduction

Low-quality diet is now known to be the main risk factor (exceeding smoking and high blood pressure) for morbidity and mortality from chronic non-communicable diseases^(1)^. Adequate nutrition is critical for healthy growth and development in the early years of life^(2)^. Inadequate nutrients’ intake can harm the nutritional status in childhood and later ages^(3)^. Brazilian data from a systematic review published in 2015 found that the food consumption of school-age children was marked by high frequencies of inadequacy in nutrients’ intake, mainly iron (22–40 %), vitamin A (20–59 %) and zinc (20–53 %)^(4)^. This recognition has encouraged studies to assess dietary intake and to improve methods for estimating individuals’ diet^(1)^. Dietary assessment in childhood is important, since it is a stage of life in which eating habits are developed, crucial for the promotion of healthy eating throughout life^(2)^. However, assessing schoolchildren's dietary intake is not simple, especially because their cognitive skills may not be fully developed. Depending on their age, children's ability to identify and remember the foods they have eaten and to describe cooking or other food preparation methods is limited^(1,5,6)^. There is also difficulty in estimating the amounts consumed^(7)^.

Dietary assessment methods are subject to systematic errors, affecting the estimated validity^(8)^. Some memory-dependent tools, such as the 24-hour recall (24-HR) and food-frequency questionnaire, are more subject to underreporting than others. On the other hand, the food record (FR) can change eating habits depending on the evaluation^(9)^. The recommended methods for assessing children's dietary intake vary according to age and the person reporting^(10,11)^. A systematic review found that the 24-HR reported by parents of children aged 4–11 years obtained higher accuracy when compared to double-labelled water^(10)^. Studies indicate that the 24-HR with the automated multiple-pass method (AMPM) using a technological tool is the best option for this age group^(12,13)^. However, direct observation and/or the FR with or without weighing has often been used to assess children's food dietary intake in school^(10,13–16)^.

Recent decades have seen the advent of new technologies for dietary assessment^(17)^. Studies have demonstrated the advantages of these technological tools in relation to the collection of data in paper format^(18,19)^: optimisation and real-time availability of results^(20)^; reduction of costs and time in data collection^(21)^; improvement of participants’ motivation in the interviews; and mainly minimisation and prevention of errors associated with dietary report, thus improving the estimates’ validity^(22)^.

Systematic reviews published in the last 5 years on the development or validation of technologies^(21,23,24)^ identified twelve studies on the development of technologies to investigate schoolchildren's diet^(16,25–39)^. Web-based 24-HR is the most frequently applied technology in this age group^(27,30–33,35,37)^, but tools have also been developed that are based on mobile devices such as smartphones and tablets^(26,36,38,39)^. Most of these technologies were developed for North American and European schoolchildren^(16,25–27,30–34,37–39)^. These systematic reviews found only one technology that was developed and validated in Brazilian schoolchildren, consisting of a qualitative dietary assessment of children aged 7–10 years through a web-based questionnaire on the previous day's diet^(28,29)^, hindering the accurate estimation of food amount and energy and nutrient intake. We also found an offline mobile app for tablets used in the 2019 Brazilian National Survey on Child Nutrition (ENANI), which investigated dietary intake in children under 5 years of age^(40)^. However, older children are known to have more autonomy over their food intake. So, the children's participation in food intake assessment in the school environment is essential, aspects that have not been investigated in other Brazilian technologies^(28,29,40–44)^. Furthermore, the use of technologies developed for different age groups^(41,44)^ to school-age children is limited.

Developing a technological tool that allows investigating the food consumption of Brazilian school-age children in a standardised and automated is important. It is crucial to know food intake in different children's environments, estimate children's diet qualitatively and quantitatively, and capture the cultural diversity of their food intake. The present study design was descriptive and aimed the development of a mobile app to assess Brazilian schoolchildren's dietary intake.

## Methods

### Description of the CADE app

CADE (*Consumo Alimentar no Domicílio e na Escola* [Food Consumption at Home and at School]) (Brazilian National Institute of Industrial Property, registration number: BR512021001151-1) is an app for mobile data collection devices (MDCD), i.e. smartphones and tablets, available on the Android platform for downloading via the Play Store, with online functionality, allowing research to assess the diet of Brazilian schoolchildren 4–9 years of age.

The CADE technology assesses the child's entire dietary intake, since it allows estimating food consumption in all dietary environments in which children eat, mainly at home and school. The app also has an option for recording previously collected dietary data.

### Development of the CADE app

#### Selection of food database, household measurements and photos of food portions

The CADE database consisted of food items ranging from simple foods and beverages to complex recipes from the 2017–18 Brazilian National Dietary Survey (INA – a national survey that assessed food consumption through two 24-HR in a probabilistic sample of individuals 10 years and older)^(45)^; the 2019 National Survey on Child Nutrition (ENANI – a nationwide survey that assessed diet through one 24-HR in a probabilistic sample of children under 5 years of age)^(40)^; menus offered in public schools across the country prepared by the technical team of the National School Feeding Program (PNAE); and the food and beverage databases of Brazilian regional surveys that assessed schoolchildren's dietary intake^(46–50)^.

Household measurements were used to quantify dietary intake, specific for each food item. The measurements were mostly extracted from the 2017–18 INA database^(45)^ and complemented by digital photos of measurements used specifically for children in the ENANI database^(40)^. Official authorisation was obtained to use digital photos of children's household measurements and food portions available in the Brazilian Manual of Child Food Portion Quantification, developed specifically for the ENANI survey^(51)^.

#### Functionalities

The dietary assessment method and the app's technological tools were chosen based on a literature review of existing tools^(15,25,27,40,52)^ and created with the collaboration of two software developers as detailed below.

The app allows two types of access: (1) by the administrator, who accesses a platform that allows the creation of user accounts by the definition of login and password. The administrator provides the user's access through a login and password. They are also responsible for performing a simple registration of the child, generating three 6-digit numeric codes in which the sixth and last digit are specific to the interface that the user completes on the child's dietary data (home, school or previously collected data) and (2) by users, who record the children's dietary data.

**Fig. 1.**
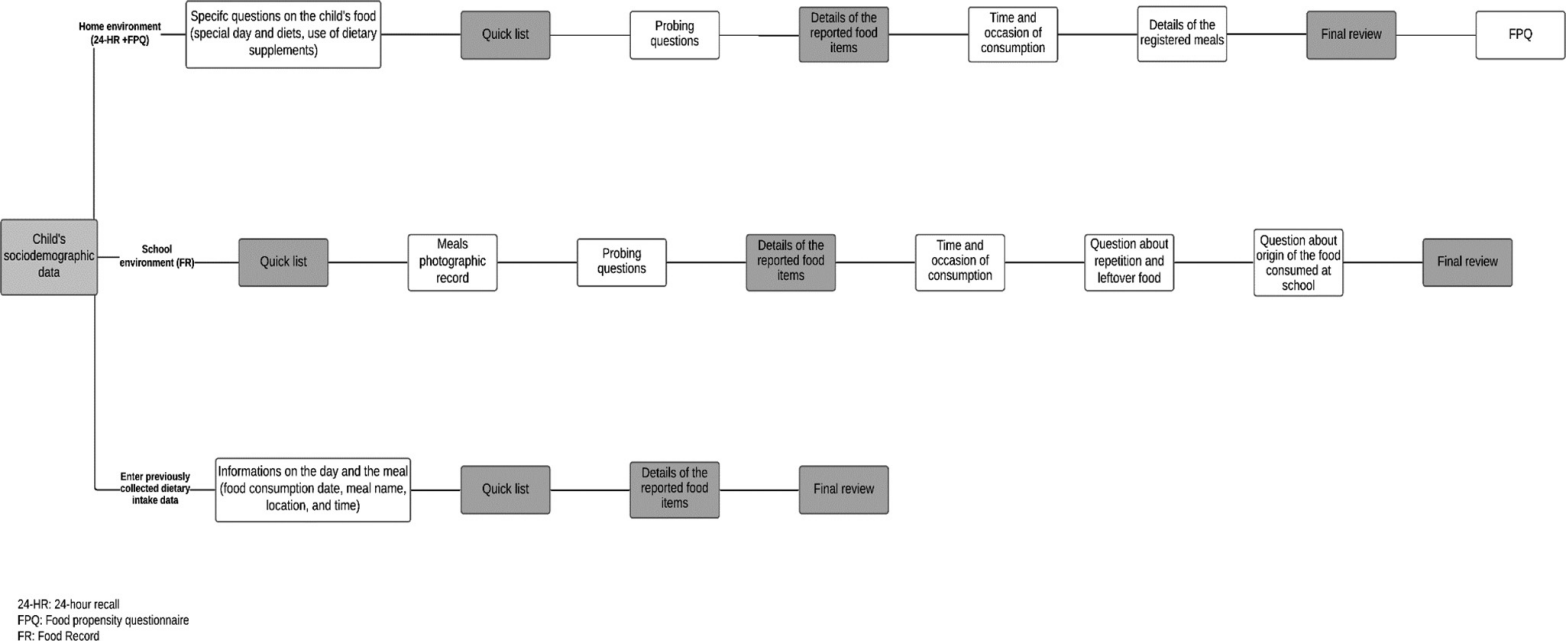
Flowchart of steps for use of the CADE app in the school and home environments and for entering previously collected dietary data.

The dietary assessment method was chosen specifically for each food environment, namely school and home, the latter including all food consumed inside and outside the home except foods consumed at school. The 6-digit numeric code allows merging data from both environments, aggregating dietary data from the same day of the child's food consumption. The child's dietary report at school is initiated by an adult aware of the child's dietary intake inside school (e.g. teacher, school staff or field researcher) using a food record (FR). On the day following the school dietary intake report, the child's dietary intake at home is recorded by parents or guardians through a 24-HR. The app also allows recording the child's dietary data that have been collected previously on paper or another medium, allowing the automation and standardisation of dietary data. Fig. 1 displays a flowchart showing the stages of dietary assessment in the app's three interfaces.

Regardless of the type of food environment, when users open the app, they must enter their login and password, previously provided by the administrator. After entering the app, they begin the report by providing the child's numerical code in a specific environment for which they are reporting the dietary intake. The child socio-demographic data including name, sex, age and place of birth are requested after accessing the child's code.

In the home environment, respondents complete the child's demographic data and answer five questions related to dietary habits: whether the reported dietary intake refers to an unusual day (with six options: holidays, travel, vacations, illness, fasting [including religious] or ‘no’); whether the child is on a special diet (ten options: lacto-ovo vegetarian, vegetarian/vegan, lactose-intolerant, egg-, milk- or gluten-allergy, phenylketonuria, diabetes, religious diet or ‘no’); what the child (or the child's parent or guardian) uses to sweeten foods or beverages consumed by the child (four options: only sugar, only sweetener, both sugar and sweetener or neither); whether the child (or the child's parent or guardian) adds salt to the child's meals (yes or no); and the frequency of seven dietary supplements (calcium, iron, multivitamins, vitamin C, vitamin D, vitamin B and omega-3/fish oil). The questions were based on the 2017–18 INA survey^(45)^, adapted to schoolchildren's dietary habits.

After these questions, the app proceeds to the 24-HR, applying the multiple-pass method^(53)^: (1) Quick list: entry of all foods and beverages consumed by the child on the previous day. Filters were created to facilitate searching foods that appear with their names repeatedly in the database, for example ‘rice’. The app also allows the inclusion of new food items that are not in the app's food database. (2) Probing questions: respondents answer questions on overlooked food items such as candy, beverages or sweets. However, these questions are answered by the children themselves when they are 8 years or older. (3) Details of food items: ways of preparing food; questions on extra food ingredients added to the reported foods (such as ketchup on hamburger); type of household measurements used to serve food or standard food units consumed. The latter was used to quantify dietary intake and included digital photos of household measurements (such as cups, spoons and bowls) and standard food portions usually consumed by children^(51)^. (4) Time and occasion of consumption: for each food item reported, the respondent must inform the name and time of the meal. (5) Details on the recorded meals: the respondent informs the place where the child ate the meal, indicating whether the child was engaged in any other activity while eating. (6) Final review: the respondent answers two questions to verify overlooked food items and double-checks all food items already reported, which are: whether child drank something between meals, considering any drink (including water), and whether he or she ate anything between meals, considering any food, even in small amounts, such as candy, cookies and snacks. (7) Food Propensity Questionnaire (FPQ): the respondent completes a food propensity questionnaire (FPQ) to inform the frequency of the child's consumption of food items in the previous month (Fig. 1), with five frequency options ranging from ‘never’ to ‘6–7 days a week’. The FPQ consisted of 38 food items distributed into seven food groups organised by their similarity and importance in the diet quality, such as grains, fruits, sausages and seafood.

In the school environment, the respondent accesses the app every time the child is eating a meal, to report the child's food intake through an FR. Dietary assessment follows the same steps that the respondent performs in the home environment: quick list, probing questions, details of food items, time and occasion of consumption and final review. However, an additional step after completing the quick food list allows the respondent to take a photo of the child's meal or food intake. The respondent can also inform whether the child had ‘seconds’ of the food (i.e. repeated the portion) or if there was any leftover meal or food on the plate. The food's origin can be recorded (whether food comes from school lunches, whether food was brought from home or bought at the school canteen). Another specific feature of the school environment is that the respondent can activate notifications from the app at the end of each child's meal report. This activation sends messages on the user's cellphone or tablet every 2 h, so that they do not forget to access it to report meals that the child eats at school.

The respondent can also use the app to enter previously collected dietary intake data. Some steps completed in the dietary data are the same as those used to assess dietary intake at school, except for the use of technological tools such as photos of the meal, digital photos that assist quantification, questions on extra food ingredients added to reported foods, and other activities performed while eating (Fig. 1).

### Managing files in the app

The app allows exporting two files related to data obtained in the school and home environments and a third file related to previously collected data. The tool's data files (.csv extension) are: (1) child's socio-demographic data from the home and school environments, such as age, gender, grade and school shift; (2) dietary intake at home and school; and (3) dietary intake from previously collected data. This latter file is only generated when dietary data are not collected directly at school or home. The exported files for the school and home environments can be ‘linked’ by the child's code, allowing analysis of the child's dietary intake on the same day.

### Data analysis

A descriptive analysis by absolute and relative frequencies was performed for characterisation of the app's database and description of its functionalities using the R Studio software, version 4.0.3.

### Ethical issues

The research project was approved by the Institutional Review Board of the Sergio Arouca National School of Public Health – ENSP/FIOCRUZ (CAAE 58831516.0.0000.5240).

## Results

The unique questions in the home environment of the CADE app concern the performance of activities during meals, with five options: use of smartphone or tablet, videogames, watching TV, use of computer or laptop and none of the above, and a FPQ consisting of thirty-eight food items distributed in seven food groups organised in blocks according to their similarity and importance in the quality of the diet, such as grains, fruit, vegetables, sausage, seafood, salty snacks, and cakes and cookies, with five options for frequency of consumption, varying from never or less than once a month to 6 or 7 days a week.

Four unique questions or features were included in the school environment: possibility of photographing the child's meals; report of leftover food with four semi-quantitative options: ‘half the meal left over’, ‘1/3 left over’, ‘almost everything left over’ and ‘almost nothing left over’; report and quantification of repetition of food items (‘seconds’); and receipt of notification to access the app to record a new meal. The remaining questions are similar in both the home and school environments.

The app features a sequence of four probing questions to detect possible overlooked foods. An interesting feature is that the app recognises the child's age at which the parent/guardian is reporting dietary intake. If the child is 8 years or older, these probing questions are addressed to the child. As already shown in international studies^(14,16,54)^, from 8 years of age onwards, children are expected to have more autonomy in their food choices and to recognise food intake and assist the parents’ report.

The CADE app's database contains 2125 food items, of which approximately 84 % were foods in the INA survey 2017–18^(45)^, 11 % from the database contained in the ENANI mobile app^(40)^, 5 % from the menus of the National School Meals Program and less than 1 % from the databases of Brazilian regional surveys. The list includes foods commonly consumed by the Brazilian population, with an emphasis on Brazilian schoolchildren's eating habits, including regional foods.

**Fig. 2.**
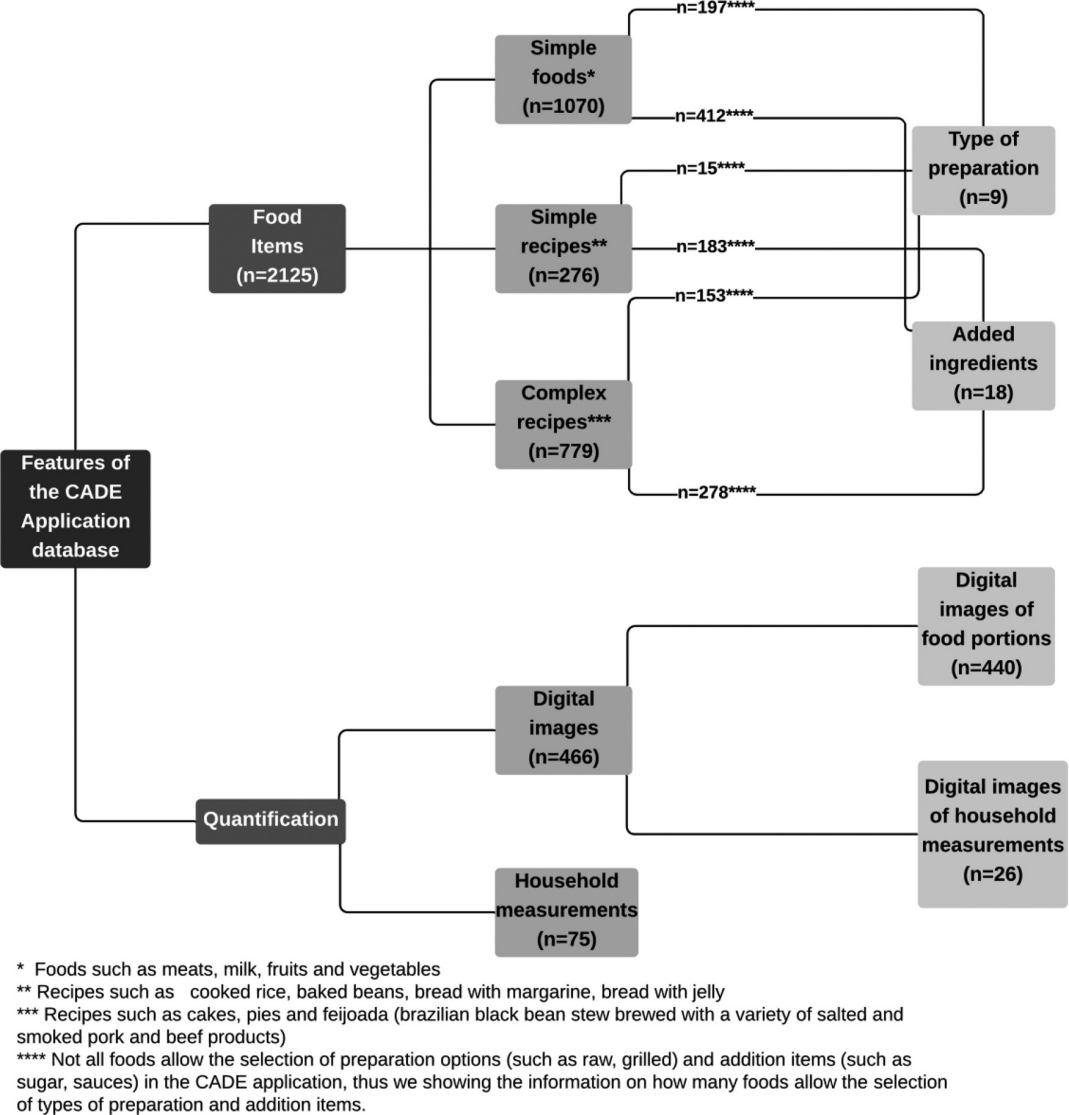
Main features of the CADE app database.

More than half of the food items list represents simple foods, such as meats, fruits and vegetables, while approximately 37 % of the database included more complex recipes such as cakes, pies and feijoada (a traditional Brazilian pork-and-beans dish). A total of nine types of food preparations such as raw, roasted and cooked with fat were available. Among the eighteen options of additional ingredients, the respondent can choose among items to sweeten food or beverages (like sugar, honey, molasses), sauces (ketchup, mustard, mayonnaise), and ingredients used by children in combination with other foods, such as granola and oatmeal. This functionality for selecting additional items is qualitative. The options for selecting the type of preparations and additional ingredients vary according to the reported food item, and not all foods allow selection of the type of preparation and additional items. Approximately 38 and 18 % of simple foods allow selection of additional ingredients and type of preparation, respectively (Fig. 2).

The quantification forms were specific to the food item and consisted of 75 household measurements (e.g. mug, glass, bowl, ladle, plate) and 466 digital images, 440 of which are digital images of 296 food portions, to facilitate reporting the amounts consumed and to minimise measurement errors with the app^(55,56)^. The size of these food portions was based on children's usual dietary intake^(51)^. A total of twenty-six digital images of four types of household items were also available, mostly used specifically by children, such as children's spoons and bowls and measures of infant formulas or supplements (Fig. 2). Such digital photos of household measurements allow estimating quantities such as ½, 1/3, 1 + 1/2, allowing more accurate quantification (Supplementary Fig. S1).

During the detailed recording of meals in the home environment and in the interface for entering previously collected dietary data, the app provides eight predefined meal options, namely breakfast, brunch, lunch, afternoon snack, dinner, supper, evening snack and late-night snack, and eight options for consumption places, such as home, home delivery, restaurant/snack bar (self-service/pay-by-weight/all-you-can-eat), restaurant (other type), recreational/social places (church, cinema, party venue), other people's homes (grandmother, friend, etc.) and on the street (food purchased from street vendors). However, details of meals in the school environment consist solely of identifying the origin of the food consumed at school, such as school lunches, food consumed in the cafeteria or food brought from home.

The final stage of the report is a review of food items and meals, plus two final questions on overlooked food items. Once the dietary intake is recorded in both environments (home and school), the app allows exporting the two files referring to the child's socio-demographic data and dietary intake at school and home.

A user's manual was developed for the app^(57)^, along with informational and demo videos for each food environment^(58)^.

## Discussion

The CADE app was based on mobile devices developed to collect dietary data from Brazilian schoolchildren using the FR and multiple-pass 24-hour recall combined with a FPQ, as well as allowing the entry of previously collected dietary data. The app was designed to standardise, automate and minimise costs and errors in the assessment of schoolchildren's dietary intake. The CADE app evaluates the school meals and integrate them with food intake at home. Moreover, the app allows to select food and household measurement digital photos to capture more accurately the amount consumed. We believed that underreporting can be minimised with this design, considering the resources that facilitate the amount consumed report and complete child's diet evaluation by investigating different consumption places^(59)^. Some new technological features compared to usual Brazilian technologies^(28,29,40–44)^, such as taking digital photos of food or meals and sending notifications in real time are only possible using online mobile devices.

The option to develop a self-report tool was mainly to make data collection feasible in two different environments, school and home. In addition, food record, the dietary assessment method in the school environment, is usually applied in self-report format. An international study showed that, compared to interview-based 24-HR, 70 % of 1081 North American adults preferred self-reported 24-HR using ASA24 (Automated Self-Administrated 24-h Recall), in all demographic groups consulted^(33)^.

Regarding the methodological resources, we opted for the 24-HR applied with the multiple-pass method accompanied by a FPQ in the home environment and the FR in the school environment, both reported by an adult answering for the child in their respective places. 24-HR administered through multiple pass is internationally recognised as the best method for dietary assessment in different age groups^(27,60,61)^. The combination of 24-HR and FPQ has been recommended to improve the estimate of the usual intake of episodic food^(62–64)^. Some studies have already reported good results with the use of a FPQ for dietary assessment of children^(65,66)^.

Meanwhile, the choice of FR in the school was based on two main issues: the need to combine the dietary data from school with the same day as those from the home environment and the assumption that whoever answers for the child's diet at school may be responsible for feeding many other children, and thus, the recall bias would be minimised by reporting child's diet in real time. In addition, FR has been proposed as the most suitable methodology for assessing young children's dietary intake^(47)^. Results from the EFCOVAL-child project showed that the FR applied for two non-consecutive days with the aid of a photographic manual for food quantification was the best method for children 4–6 years of age^(13)^.

The choice of the 24-HR and FR also assumed that quantitative dietary assessment is essential in this age group. Energy and nutrient intake, prevalence of inadequate nutrient intake, food pattern analysis and adequacy of diets are some examples that depend on quantitative data^(67,68)^.

The CADE database was developed to be comparable to databases from the Brazilian national surveys^(45,69)^. However, few technologies have been developed specifically for the Brazilian population, and even fewer use databases based on nationwide dietary intake^(40,41,44)^. The development of Brazilian technologies to assess dietary intake is incipient and recent in the country. In addition, technological advances already developed internationally such as automated recognition and quantification of foods in digital photos, the use of cameras and sensors in dietary assessment, and the use of food product barcodes in data collection has still not been described in any Brazilian technologies^(21,23,70)^.

Quantitative estimates of dietary intake have been recognised as a major source of error in self-reported dietary assessment methods^(53)^. Photographs of food portions have been identified to improve the estimation of portion sizes^(55)^; therefore, some automated 24-HR have integrated digital photo viewing into their platforms^(22,71–75)^. A recent Brazilian study using 24-HR from GloboDiet software found that digital and print photographs provided greater flexibility in interviewing individuals with less education, besides facilitating the quantification stage during the report. According to the authors, all respondents stated that it was ‘easy’ to quantify foods using photographs^(55)^. In another study with the same data stated above, the authors showed that individuals were able to properly recognise food portion sizes using print and digital photos available on a tablet. However, the authors found that among individuals with low schooling, digital photos in tablets were more subject to underestimation than print photos^(56)^.

In the CADE app, users in the school environment can take photos of the food or dish. Although the app lacks technology for automatic recognition and quantification of the photographed food, we believe that the photo may be useful in the short term, helping to manually recognise any food item that may have been overlooked in the report. Besides, in the long term, it can help produce an image database that can be used in technological innovations employing artificial intelligence and allowing the image-assisted recognition and measurement of food^(56,74–76)^.

The technology-based assessment of dietary intake has become increasingly relevant, even essential, acknowledging the need to optimise traditional dietary assessment instruments, standardise the data collection, obtain more accurate and reliable data, develop more innovative and less expensive survey alternatives, and enhance the interviewee's attention and motivation, thereby increasing the response rate^(76)^. From this perspective, technologies applied to smartphones seem attractive, since the use of these mobile devices has become increasingly popular. International studies have reported good results with the use of smartphones to assess dietary intake, demonstrating that they can be intuitive, practical and modern, thus serving as the technology of choice for participants^(77,78)^.

In 2018, 99⋅2 % of Brazilian households used the internet, with cellphones as the most widely used technological tool for this purpose, with little difference between urban and rural areas^(79)^. Despite access to the internet and the widespread use of cellphones in Brazil, individuals from lower socioeconomic strata experience limitations in the use of internet and mobile devices^(80,81)^, and even individuals from higher socioeconomic strata may display digital illiteracy in the use of these tools^(79,82)^. However, an international study has found that even in low-income countries, the electronic collection of dietary data can be a feasible alternative^(36,83)^.

The app presents some limitations, such as the recipe list in the database. The list in the app is closed, not allowing modification of recipe ingredients. This issue can be minimised with the possibility of inserting new foods and/or recipes. It is worth mentioning that only one Brazilian technological tool is capable of editing recipes: the Brazilian version of GloboDiet, which consists of an offline software that assesses food consumption through an interview-based 24-HR^(44)^. However, GloboDiet was developed for adults and is still not freely accessible. Another limitation is that the app does not integrate information on the nutritional composition and weight of the amount consumed in an automated way, which would allow it to generate instant feedback on individuals’ energy and nutrient intakes.

In addition, the app has still not been tested initially for its usability and later for its validity in dietary assessment. These evaluations will help identify the average time spent to record the dietary data, the functionalities that need to be reviewed and improved, the data quality, and the tool's applicability to specific population groups such as individuals with less schooling.

Regarding the strengths of the present study, the CADE app is the first Brazilian technological tool to assess schoolchildren's qualitative and quantitative dietary data. As it is a technology-based on mobile collection device, it may promote accessibility, ease of data collection, and cost reduction for scientific research. The tool features improve episodically food intake estimates by combining the 24-HR and FPQ methods, enhance the reporting amount consumed by integrating digital photos of food portions and household measurements, and the manual recognition of forgotten food items by photographing children's meals in the school environment.

Efforts are needed to make this technological tool available to large-scale studies: testing the app usability to improve the design, flow and content of the application based on users feedback, and testing the app usability specifically among less educated Brazilian children. After the usability tests, we intended to evaluate the validity of the app dietary information compared to other food assessment methods and biomarkers. After these steps, we believe that the CADE app can assist the standardisation and automation of schoolchildren's dietary data collection, promoting the development of studies in nutritional epidemiology and nutrition and public health, as well as in primary healthcare for children.

## Conclusion

The app design included complex flows that integrate the food consumption assessment in school and home environments and allow entering previously collected food data. Its features are innovative, such as viewing digital photos of household measurements and food portions to estimate the amount consumed and receive notifications to remind the user to report the children's food intake. Thus, providing a technological tool to assess Brazilian school children's food consumption can reduce biases, costs and time.

## Supplementary material

For supplementary material accompanying this paper visit https://doi.org/10.1017/jns.2022.25. click here to view supplementary material
