# Supplementary material for: Development of a mobile application to assess Brazilian schoolchildren's diet: CADE – food consumption at home and at school
Source: J Nutr Sci. 2022 Apr 11;11:e27. doi: 10.1017/jns.2022.25 (PMC9003633; doi:10.1017/jns.2022.25)
Supplement: Supplementary file 1 [file S2048679022000258sup001.docx]

**Supplementary Materials**


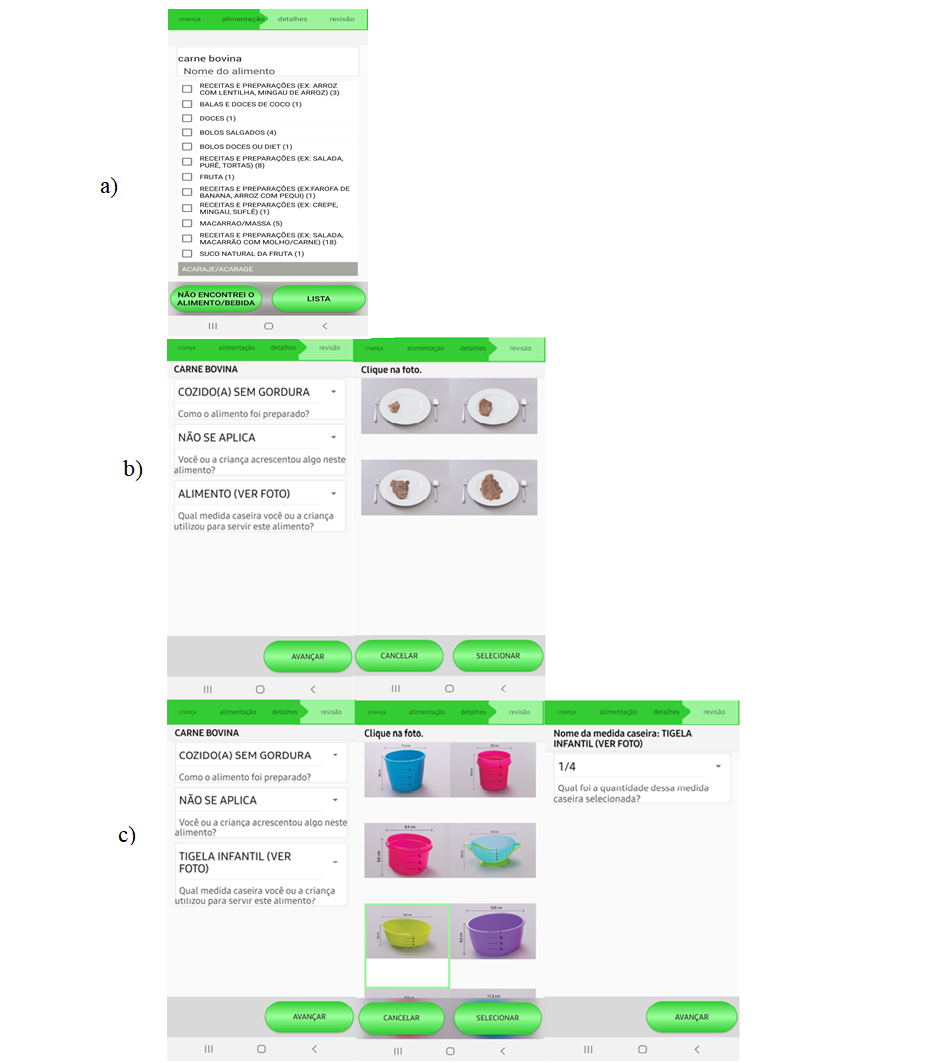
 **Supplementary Figure S1.** Quantification of a steak in the CADE application. (a) selection of beef through the CADE app food list (b) Quantification using digital images of food portions; (c) quantification using digital images of household measurements. Digital photos from ^(51)^
